# Dynamics of cerebrospinal fluid levels of matrix metalloproteinases in human traumatic brain injury

**DOI:** 10.1038/s41598-020-75233-z

**Published:** 2020-10-22

**Authors:** Karolina Minta, Gunnar Brinkmalm, Faiez Al Nimer, Eric P. Thelin, Fredrik Piehl, Mats Tullberg, Anna Jeppsson, Erik Portelius, Henrik Zetterberg, Kaj Blennow, Ulf Andreasson

**Affiliations:** 1 Department of Psychiatry and Neurochemistry, Institute of Neuroscience and Physiology, the Sahlgrenska Academy at the University of Gothenburg, Gothenburg, Sweden; 2 Clinical Neurochemistry Laboratory, Sahlgrenska University Hospital, Mölndal, Sweden; 3 Department of Clinical Neuroscience, Karolinska Institutet, Stockholm, Sweden; 4 Department of Neurology, Karolinska University Hospital, Stockholm, Sweden; 5 Department of Clinical Neuroscience, Institute of Neuroscience and Physiology, the Sahlgrenska Academy at the University of Gothenburg, Gothenburg, Sweden; 6 Department of Neurodegenerative Disease, UCL Institute of Neurology, London, UK; 7 UK Dementia Research Institute at UCL, London, UK

**Keywords:** Neuroscience, Biomarkers, Medical research, Neurology

## Abstract

Matrix metalloproteinases (MMPs) are extracellular enzymes involved in the degradation of extracellular matrix (ECM) proteins. Increased expression of MMPs have been described in traumatic brain injury (TBI) and may contribute to additional tissue injury and blood–brain barrier damage. The objectives of this study were to determine longitudinal changes in cerebrospinal fluid (CSF) concentrations of MMPs after acute TBI and in relation to clinical outcomes, with patients with idiopathic normal pressure hydrocephalus (iNPH) serving as a contrast group. The study included 33 TBI patients with ventricular CSF serially sampled, and 38 iNPH patients in the contrast group. Magnetic bead-based immunoassays were utilized to measure the concentrations of eight MMPs in ventricular human CSF. CSF concentrations of MMP-1, MMP-3 and MMP-10 were increased in TBI patients (at baseline) compared with the iNPH group (p < 0.001), while MMP-2, MMP-9 and MMP-12 did not differ between the groups. MMP-1, MMP-3 and MMP-10 concentrations decreased with time after trauma (p = 0.001–0.04). Increased concentrations of MMP-2 and MMP-10 in CSF at baseline were associated with an unfavourable TBI outcome (p = 0.002–0.02). Observed variable pattern of changes in MMP concentrations indicates that specific MMPs serve different roles in the pathophysiology following TBI, and are in turn associated with clinical outcomes.

## Introduction

Traumatic brain injury (TBI) is a complex disorder that comprises different types of pathophysiological disease processes, often resulting in life-long cognitive, behavioural and physical disabilities^1,2^. Annually, almost 70 million people worldwide are affected by TBI^3^. The traumatic impact may cause stretching and tearing of axons, leading to microtubule disruption and impaired axonal transport^4^. In severe TBI, where structural lesions are apparent and the patient is unconscious, blood–brain barrier (BBB) disruption, mitochondrial dysfunction, oxidative stress and inflammation are common pathophysiological processes^4^. Early diagnosis of brain injury severity is important to prevent or reduce the gradually evolving secondary damages to the brain and thus improve patient recovery^5^. There are several scoring systems used to assess TBI severity. These include, among others, assessments of consciousness [Glasgow Coma Scale (GCS)^6^ and Reaction Level Scale-85 (RLS-85)^7^], consciousness in combination with structural injuries [Abbreviated Injury Scale (AIS)^8^], structural injuries on computed tomography (CT) scans of the brain (Marshall-CT^9^ classification, Rotterdam-CT score^10^, Helsinki CT score^11^ and Stockholm CT score^12^) and structural injuries on magnetic resonance imaging (MRI) (e.g. Firsching MRI score^13^). Prognosis of outcome following TBI is very important for the correct estimation of medical care and treatment, resulting in research endeavours like the International Mission for Prognosis and Analysis of Clinical Trials (IMPACT) in TBI consortium, establishing independent outcome predictors^14^. The Glasgow Outcome Scale (GOS) is the most commonly used assessment (5-point) of functional outcome following TBI^15^.

The extracellular matrix (ECM) surrounds cells in the central nervous system (CNS) and occupies approximately 20% of the brain tissue volume^16^. It is a highly dynamic structure that continuously undergoes controlled remodelling. The balance between the synthesis, development and degradation of ECM components is important to ensure normal physiology^17^. However, in cases of altered expressions of matrix proteases, this homeostasis can be dysregulated, leading to various pathological conditions^17^.

Owing to its close contact with the brain, cerebrospinal fluid (CSF) is a relevant compartment for studies of biological and pathological processes that affect the brain and spinal cord. To date, there are several promising CSF biomarkers for brain tissue injury, e.g., neurofilament light (NFL)^18,19^, total tau (t-tau)^18–22^, S100 calcium-binding protein B (S100B)^19,23,24^ and neuron-specific enolase (NSE)^24^. Even though NFL, NSE and S100B as blood biomarkers are used for brain injury^25–28^, there is a growing interest in CSF measurements of these proteins, mainly due to their higher abundance in CSF compared with blood. Several studies indicate that CSF concentrations of NFL, t-tau, S100B and NSE are acutely elevated in TBI patients and progressively decrease over time^18–24^. However, still there is a great need to develop more neurochemical indicators reflecting additional pathological processes in TBI.

Matrix metalloproteinases (MMPs) belong to a multigene family of 23 extracellular endopeptidases^29^, and are involved in ECM turnover and degradation of its constituents. Changes of MMPs expression after TBI are implicated in the pathogenesis of more severe TBI^30^. For example, MMP-3^31^ and MMP-9^32^ gene knock-out mice show reduced BBB disruption, while MMP-7 and MMP-9 immunoreactivity is induced in active demyelinating human CNS lesions^33^.

MMPs can be detected and quantified in CSF and some studies have investigated their levels in CSF from TBI patients^34,35^. CSF levels of MMP-9 have been the most investigated in TBI and the studies consistently show increased CSF concentrations of MMP-9 following TBI^34,35^. Moreover, CSF MMP-9 concentrations have been shown to reflect TBI severity, with higher levels reported in patients with severe, compared with moderate, TBI^34^. Previous reports concerning other MMPs have shown increased CSF concentrations of MMP-3 in TBI patients compared with normal pressure hydrocephalus (NPH) group^35^, whereas CSF MMP-2 gave conflicting results, being either elevated^34^ or unchanged^35^ in TBI.

Thus, the most extensively investigated MMPs in TBI are MMP-2, MMP-3 and MMP-9. However, looking at wider array of MMPs is necessary to fully understand the dynamics of the individual MMPs on underlying pathophysiological processes in TBI and assist in the development of novel therapeutic targets to improve the outcome following TBI.

We hypothesized that the CSF concentrations of MMPs would be increased in TBI patients compared with the contrast group and that elevated levels would reflect the extent of damage to the ECM, and thus an unfavourable outcome following TBI. To test the hypothesis we measured concentrations of eight different MMPs, combing unexplored in CSF MMPs (MMP-1, MMP-7, MMP-10 and MMP-12) and previously investigated MMPs (MMP-2, MMP-3 and MMP-9) in relation to TBI and iNPH, as the latter MMP set showed a potential to reflect the pathological processes in CSF.

## Results

### MMP CSF levels

#### Comparisons of MMP CSF levels between TBI and iNPH patients

**Figure 1:**
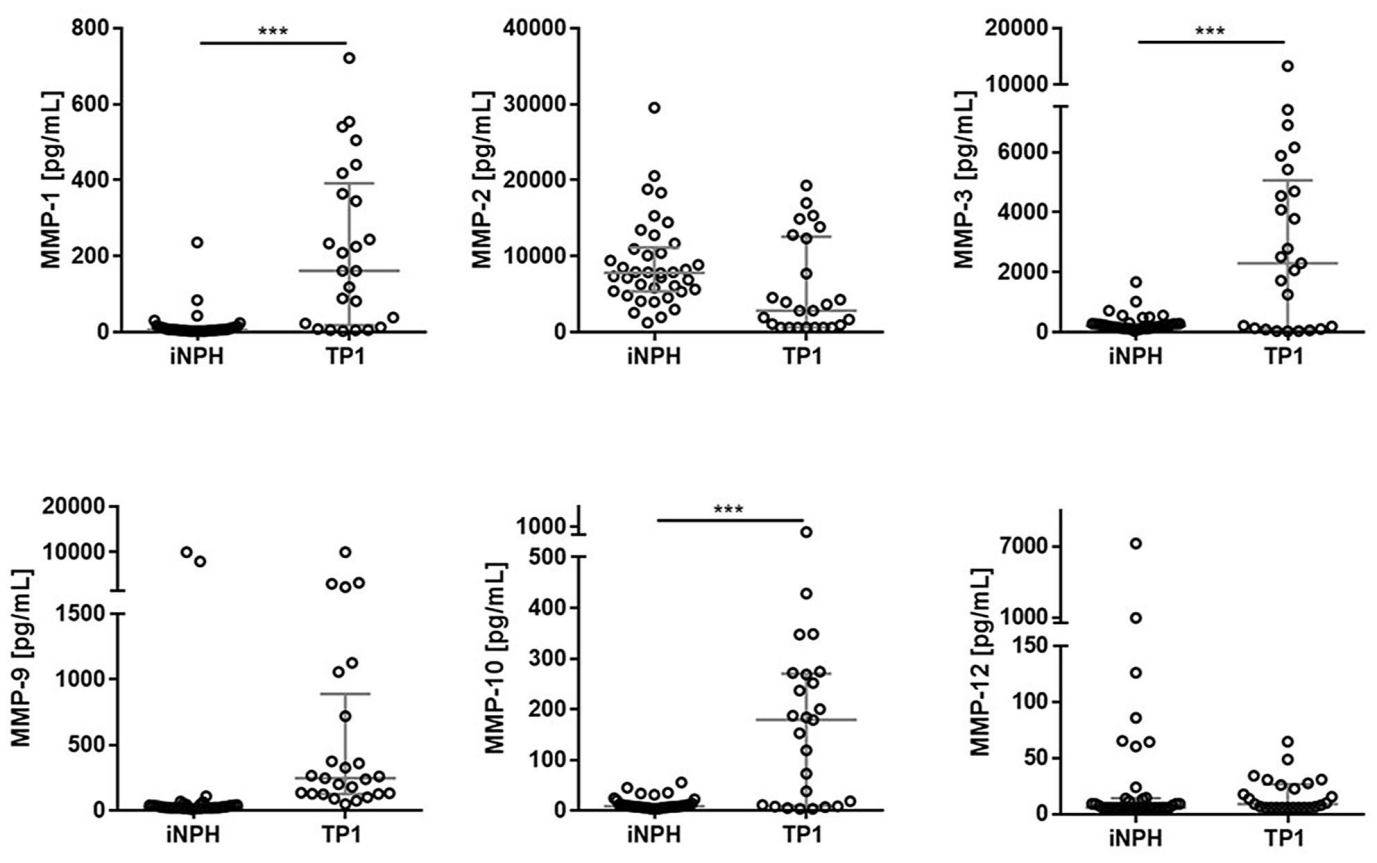
CSF concentrations for the measured MMPs for the iNPH and TBI groups. The horizontal lines represent the median and interquartile ranges. Statistical significance in CSF MMP concentrations between TBI group (time point 1; TP 1) and contrast (iNPH) group: ***p < 0.001. Analysis of covariance was used to examine the differences between the groups, accounting for the effect of age. N (number of patients): n = 38 for iNPH, n = 25 for TBI (TP1).

Since there was an age difference between iNPH and TBI groups (Table 1), an age adjustment is needed. When corrected for age, CSF MMP-1, MMP-3 and MMP-10 concentrations were elevated in TBI patients at baseline (TP1) compared with iNPH patients (p < 0.001), while CSF MMP-2, MMP-9 and MMP-12 concentrations did not differ (Fig. 1). When age adjustment was not applied, CSF MMP-1, MMP-3, MMP-9 and MMP-10 concentrations were significantly elevated in TBI group compared with iNPH patients (p = 0.001–0.01), while CSF MMP-2 showed a different change, being significantly decreased in TBI group compared with iNPH patients (p = 0.005). MMP-7 was excluded from the analysis due to its high inter-variability (CV = 27%). MMP-13 was not detectable in CSF.

**Table 1:** Participant demographics.

Characteristic	TBI (n = 33)	iNPH (n = 38)
**Gender, n (%)**		
Male	24 (73%)	28 (74%)
Female	9 (27%)	10 (26%)
Age, median (interquartile interval)	53 (42–63)	67 (59–70)
Outcome (favourable/unfavourable)	52%/48%	
**Trauma severity scoring, median (interquartile interval), % severe TBI**		
GCS	4 (3–9), 73%	
AIS	5 (4–5), 87%	

Outcome prediction is dichotomized as unfavourable (GOS = 1–3) and favourable (GOS = 4–5).

A total score of 3–8 for GCS or 4–6 for AIS indicates severe TBI.

*AIS* abbreviated injury scale, *GCS* Glasgow Coma Scale, *iNPH* idiopathic normal pressure hydrocephalus, *TBI* traumatic brain injury.

#### Longitudinal levels of MMP CSF levels in TBI patients

**Figure 2:**
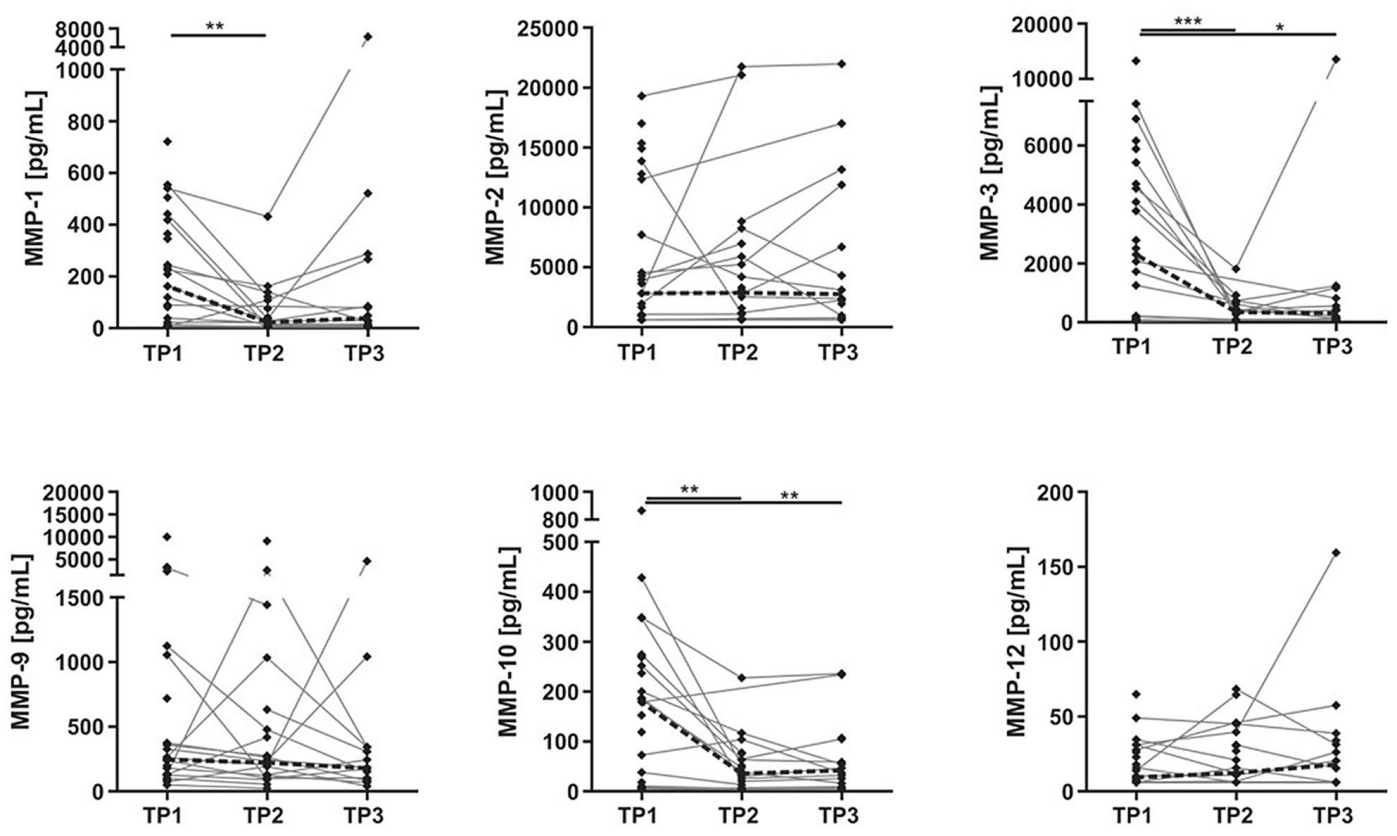
Repeated measurements of MMPs in TBI patients at three time points (TPs). Repeated measures of six different MMPs (MMP-1, MMP-2, MMP-3, MMP-9, MMP-10 and MMP-12) in CSF, obtained at three TPs following TBI. Statistical significance: *p ≤ 0.05, **p ≤ 0.01, ***p ≤ 0.001. The differences between the longitudinal measurements were analysed using linear mixed effects model. The dashed lines represent the longitudinal median changes.

Longitudinally, CSF concentrations of MMP-1 decreased significantly from TP 1 to TP 2 (p = 0.01), while MMP-3 and MMP-10 decreased from TP 1 to TP 2 and from TP 1 to TP 3 (p = 0.001–0.04) (Fig. 2). There were no intra-individual changes in CSF MMP-2, MMP-9 and MMP-12 concentrations in longitudinal samples (Fig. 2).

#### Associations between MMP CSF levels and outcome

**Figure 3:**
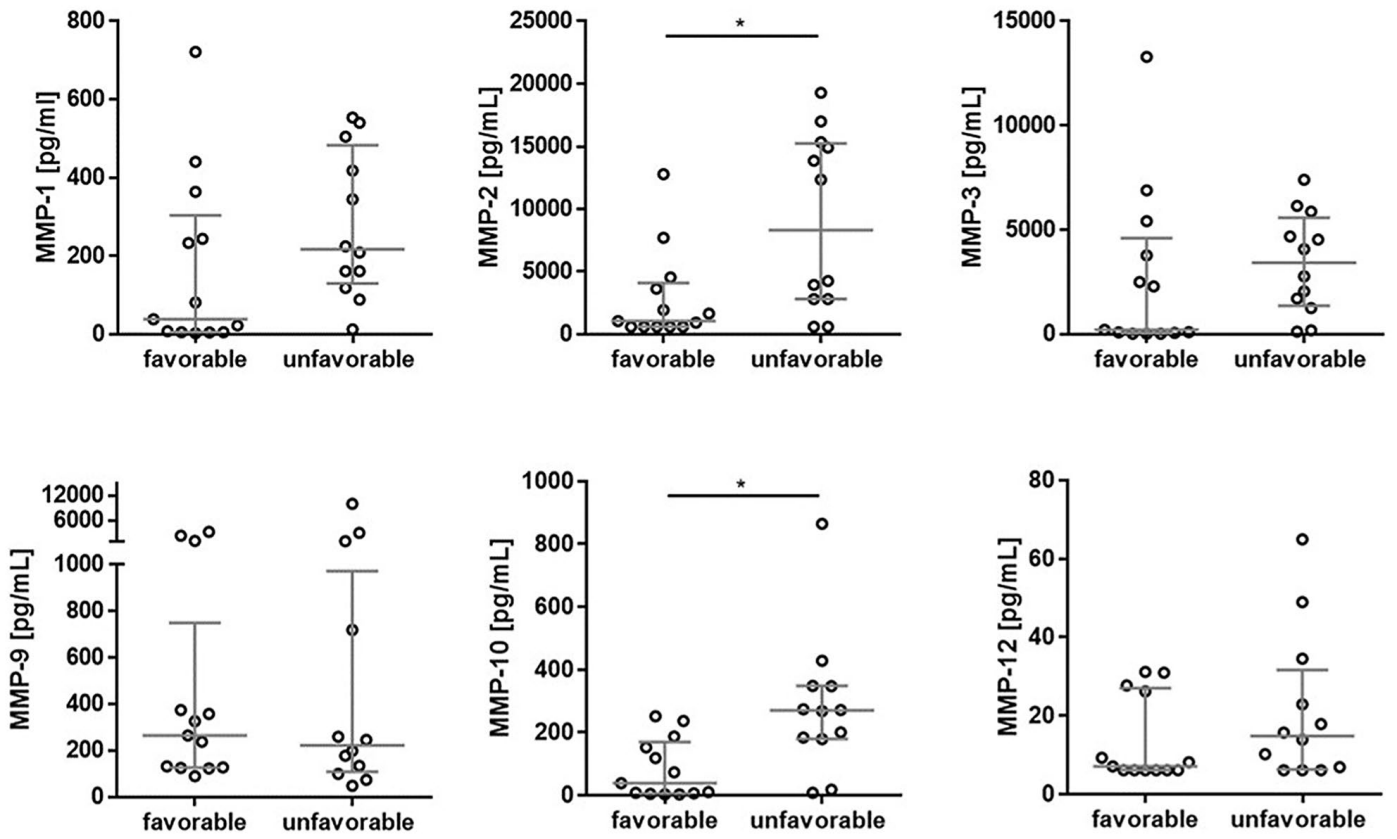
Association between MMPs in CSF and outcome in TBI patients (at time point 1). The horizontal lines represent the median and interquartile ranges. Statistical significance in CSF MMP concentrations between favourable (GOS = 4–5) and unfavourable (GOS = 1–3) outcomes at the baseline following TBI: *p ≤ 0.05. Analysis of covariance was used to examine the differences between the groups, accounting for the effect of age. N (number of patients): n = 12 for favourable, n = 13 for unfavourable.

Nearly half of the TBI patients (48%) included in this study were assessed with unfavourable outcome at 12 months after trauma (Table 1). The CSF MMP-2 (AUC = 0.77, p = 0.02) and MMP-10 (AUC = 0.85, p = 0.002) concentrations were significantly increased in TBI patients with unfavourable outcome compared with favourable (Fig. 3, Table 2). For MMP-1, MMP-3, MMP-9 and MMP-12 there was no statistical difference in CSF concentrations between outcome groups (Fig. 3). Even though CSF MMP-1 and MMP-3 concentrations were not significantly different between the groups, they were increased in the unfavourable group at a trend level (AUC = 0.68–0.71) (Table 2). There are two clear subgroups in the unfavourable outcome of CSF MMP-2 measurements, where patients with higher concentrations were older (median = 65) compared with patients with lower concentrations (median = 50), that in turn are at the same age as patients in favourable group (median = 50). CSF MMP-9 and MMP-12 also exhibit two subgroups in favourable and/or unfavourable outcome. However, there is no clear age-dependence within these subgroups.

**Table 2:** Summary of Receiver Operating Characteristic (ROC) analysis, favourable vs unfavourable outcome following TBI, of MMPs and brain injury biomarkers in CSF.

MMP	AUC	Cut off (pg/mL)	Sensitivity (%)	Specificity (%)
MMP-1	0.705	85.3	91.7	61.5
MMP-2	0.769*	2380	83.3	69.2
MMP-3	0.679	114	100	46.2
MMP-9	0.468	133	75.0	38.5
MMP-10	0.846*	166	83.3	76.9
MMP-12	0.635	9.75	66.7	69.2

Biomarker	AUC	Cut off (ng/mL)	Sensitivity (%)	Specificity (%)
NFL	0.969**	3480	100	87.5
S100B	0.766	22.8	100	50.0
NSE	0.672	43.0	100	50.0

Statistical significance in the CSF analyte concentrations between favourable and unfavourable outcomes in TBI: *p ≤ 0.05. Receiver Operating Characteristic analysis was performed to predict the unfavourable outcome following TBI.

*AUC* area under the curve, *MMP* matrix metalloproteinase, *NFL* neurofilament light, *NSE* neuron-specific enolase, *S100B* S100 calcium-binding protein B.

#### Association between MMP CSF levels and extracranial trauma

Five TBI patients having the baseline measurement (TP1) also suffered from extracranial trauma (Supplementary Fig. 1). There was no difference in CSF MMPs concentrations between patients with additional extracranial injuries and those with intracranial injuries only (Supplementary Fig. 1).

#### Interactions between CSF MMP levels and clinical variables

**Figure 4:**
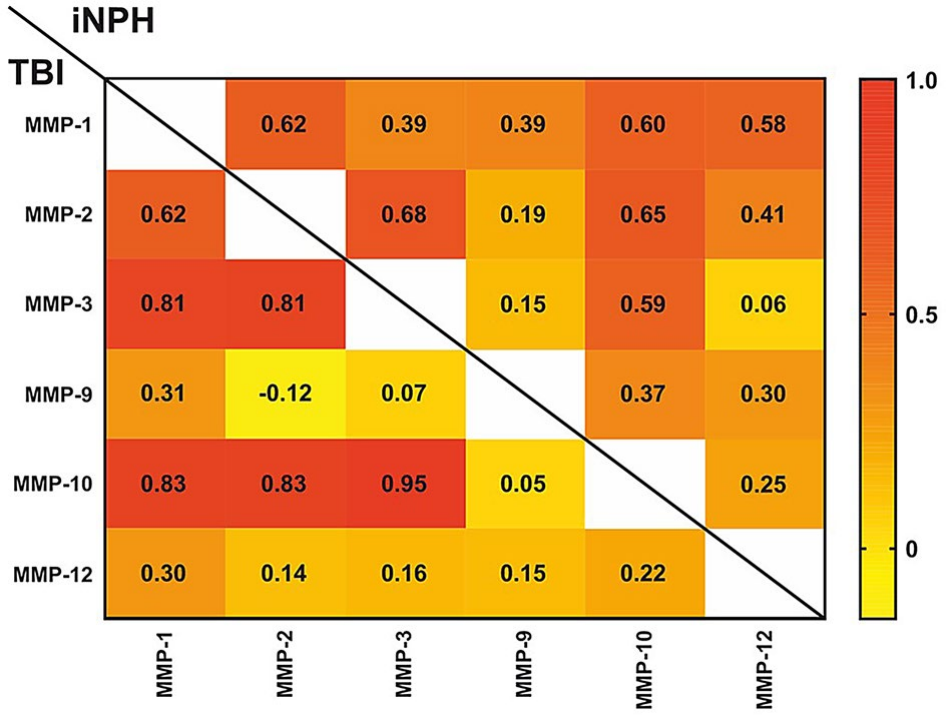
Correlation matrix between the CSF concentrations of MMPs within TBI and iNPH groups separately. The correlation matrix displays Spearman’s correlation coefficients (rho) between the CSF MMP concentrations in TBI (lower-left part) and iNPH (upper-right part) groups. The darker and more red the box, the closer the correlation is to positive 1.

**Figure 5:**
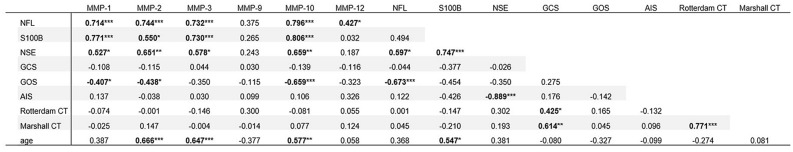
Correlation matrix between the CSF concentrations of MMPs and biomarker for brain injury and severity/outcome scores. The correlation matrix contains Spearman’s correlation coefficients (rho) between each variable. GOS is used as an ordinal scale (GOS = 1–5) in correlation matrix. Statistical significance: *p ≤ 0.05, **p ≤ 0.01, ***p ≤ 0.001. *AIS* abbreviated injury scale, *CT* computerized tomography, *GCS* Glasgow Coma Scale, *GOS* Glasgow Outcome Scale, *MMP* matrix metalloproteinase, *NFL* neurofilament light, *NSE* neuron-specific enolase, *S100B* S100 calcium-binding protein B.

The concentrations of MMP-1, MMP-2, MMP-3 and MMP-10 correlated with each other in the TBI group (AUC = 0.62–0.95, p < 0.001) (Fig. 4). These MMPs also correlated significantly with each other in the iNPH group, but correlations were weaker (0.39–0.65, p = 0.001–0.02) (Fig. 4). There was no or very weak correlation between MMP-9 or MMP-12 and other MMPs in the TBI group (AUC = − 0.13 to 0.31) (Fig. 4). In the iNPH group, MMP-12 correlated with MMP-1 and MMP-2, while MMP-9 correlated with MMP-1 and MMP-10 (AUC = 0.37–0.58, p = 0.001–0.02). However, MMP-1, MMP-2 and MMP-10 correlated negatively with GOS (rho = − 0.41 to − 0.66, p = 0.001–0.04) (Fig. 5), thus higher MMP levels indicated a more unfavourable long-term functional outcome. None of the MMPs correlated with any of the severity scores such as GCS, AIS, Rotterdam CT or Marshall CT (Fig. 5).

### Other biomarkers for brain injury

The ROC analysis showed an excellent ability for the CSF NFL biomarker to discriminate between favourable and unfavourable outcome groups (AUC = 0.97, p = 0.002) (Table 2). Other brain injury biomarkers, CSF S100B and NSE, showed good, but not significant, separation between the two outcome groups (AUC = 0.67–0.77).

NFL correlated significantly with all MMPs, except MMP-9 (rho = 0.43–0.80, p ≤ 0.05) (Fig. 5). S100B and NSE correlated significantly with most of the MMPs, except MMP-9 and MMP-12 (rho = 0.53–0.81, p ≤ 0.05) (Fig. 5).

## Discussion

The results indicate significant changes in the CSF concentrations of several MMPs following TBI, where main findings are that CSF MMP-1, MMP-3 and MMP-10 concentrations are increased in TBI patients compared with the contrast group and gradually decrease with time following brain injury. To our knowledge, this is the first report describing MMP-2 and MMP-10 as potential CSF biomarkers for clinical outcome prediction in TBI.

Biochemical processes implicated in TBI are complex and depend both on type of trauma and individual factors. While primary injury processes are closely associated with the type of trauma suffered and therefore difficult to avoid, subsequent secondary tissue disease processes represent a potential target for therapeutic interventions.

MMPs are known to be involved in secondary brain injury cascades that results in BBB disruption, haemorrhage and neuroinflammation^36^. However, the mechanisms and functional consequences of these proteases following TBI are largely unexplored. An improved understanding of the dynamics of the individual MMPs following brain injury would shed light on their role in TBI and potential as targets for therapeutic interventions.

The variability in the dynamic changes among MMPs following TBI and between the groups might indicate that various types of MMPs not only reflect different disease specific pathophysiological mechanisms but also play different roles in the same disease.

For instance, elevated CSF MMP-1, -3 and -10 concentrations in the TBI group could reflect increased degradation of proteoglycans in this group and, hence, stimulate axonal recovery and synaptogenesis to create an open, accessible environment for reconnection in the injured brain. While some MMPs might be beneficial in restoring the homeostatic ECM following the brain injury, others can impose detrimental effects^37^. For instance, the increase of MMP-10 in TBI is also associated with unfavourable outcome following TBI. However, it is also possible that MMP elevation is initially beneficial, but when over-increased it becomes deleterious^37^. Moreover, it cannot be excluded that increased MMPs levels in CSF might be rather a result of their increased leakage from the brain tissue into the CSF in TBI than their increased production following TBI, or both.

As CSF MMP-2 and -12 levels are comparable in both the iNPH and the TBI groups, they might be more specific to the pathological processes that are common for iNPH and TBI, e.g., neuroinflammation, rather than stimulation of neurite outgrowth.

The differential association of individual MMPs with specific pathological processes in TBI, could help to identify the targets for therapeutic interventions, depending on the observed pathology. However, it cannot be excluded that MMP production is a side effect of more complex processes in TBI, and therefore targeting them will not necessarily improve the outcome. Thus, further studies are needed to evaluate the role of individual MMPs in the pathophysiology of TBI. At this point, the CSF MMP-2 and MMP-10 concentrations presented in this study are proposed to improve the quality of clinical outcome prediction, and help clinicians to understand the patient’s condition and proceed with suitable treatment.

Prior studies have documented increased expression of several MMPs in the context of experimental models of TBI as well as in humans (reviewed in Ref.^36^). Under normal physiological conditions, MMPs have been shown to be expressed at low background levels that increase under pathological conditions^36^. The exception is MMP-2, which is continuously expressed in the brain^38^.

This study shows that MMP-1, MMP-3 and MMP-10 were increased in the TBI group compared with iNPH, representing a more chronic disease condition with less intense pathophysiology. Even though MMP-9 showed a clear trend to be increased in the TBI, lack of significant differences between iNPH and TBI groups might be partially explained by its association with age (since the difference between the groups lost its significance after age-adjustment). It is important to highlight that the iNPH group does not represent healthy individuals. Previous studies indicate that iNPH involves several pathophysiological changes including microglia and astroglial activation as well as axonal damage indicated by elevated CSF NFL levels^39,40^. Thus, it remains unclear if MMP-2 is increased or unchanged compared with the non-pathological state in the iNPH group.

The decrease in CSF MMP-1, MMP-3 and MMP-10 over time after TBI is likely due to clearance mechanisms after an injury-induced initial increase, e.g., by endocytosis of α2-macroglobulin/MMP complexes^41^. MMP-2 and MMP-9 showed no change in longitudinal data, which is in line with a previous study^34^.

Since most of the MMPs correlate with age, adding age as a co-variate in the LMM models is necessary. However, there could be hidden confounders when adjusting for age as age itself might be associated to the type of trauma the patient suffered from, e.g. younger patients usually have more diffuse brain injury. Nonetheless, when removing age from the LMM models, the longitudinal differences in CSF MMP-1, MMP-3 and MMP-10 concentrations remained significant.

To our knowledge, this is the first report describing CSF MMP concentrations in relation to TBI outcome prediction. The results presented show that both MMP-2 and MMP-10 in CSF predict clinical outcome in TBI to a similar degree as S100B and NSE. Interestingly, there are two clear subgroups in the unfavourable outcome of CSF MMP-2 measurements. The association of CSF MMP-2 concentrations to age might explain this. The patients with unfavourable outcome and lower CSF MMP-2 concentrations were younger compared with the subgroup with higher CSF MMP-2 concentrations, but similar in age when compared with favourable group.

Although CSF MMP-9 and MMP-12 concentrations also exhibit two subgroups in favourable and/or unfavourable outcomes, there is no evident age-dependence within these subgroups. In support to that, there is no correlation between age and CSF MMP-9 or MMP-12 concentrations in the TBI group. The rest of the MMPs have even distribution within the same outcome group. However, since CSF MMP-2, -3 and -10 concentrations have significant correlations with age in the TBI group, it cannot be excluded that their levels generally increase with age, independently on outcome. Thus, the age-adjustment is necessary and was applied when investigating the group differences. Additionally, MMP-1 and MMP-3 were elevated at a trend level in unfavourable compared with favourable outcome. It is also important to note that NFL showed to be a superior marker for TBI outcome prognosis. Thus, a surrogate marker for axonal degeneration in CSF seems to illustrate the pathophysiology which is the primary determinant of outcome following TBI.

Even though MMP-1, MMP-2, MMP-3 and MMP-10 belong to different subgroups categorized based on MMP structure and substrate specificity, the significant correlations between them indicate that they might be regulated in similar ways and perhaps also exhibit related functions. Interestingly, their correlations are much higher in the TBI group compared with iNPH, suggesting that they reflect comparable biological and/or pathological processes specific to TBI. Moreover, they highly correlate to other biomarkers reflecting brain injury, i.e., NFL, S100B and NSE, suggesting that they might be suitable biomarkers for both neuronal and astroglial damage. The lack or weak correlations of MMP-9 and MMP-12 to other MMPs and to brain injury biomarkers (NFL, S100B, NSE) indicate that they might measure different pathological changes.

The findings in this study show that individual MMPs have different dynamics in TBI. This might be due to the fact that MMPs are regulated by a wide variety of factors. For example, the activity of MMP-9 is mainly controlled by tissue inhibitor of metalloproteinases (TIMP)-1, while TIMP-2 is an important inhibitor for MMP-2^42,43^. Also, the binding affinities vary for different TIMP/MMP pairs^44^. For example, TIMP-2 and TIMP-3 are weaker inhibitors than TIMP-1 for MMP-3, contrasting with their affinities for other MMPs^45^. MMPs might also have different substrate preferences, e.g., MMP-2, but not MMP-9, degrades two brain-specific extracellular matrix proteins—brevican and neurocan^46,47^. The differential regulation and substrate preferences of individual MMPs can lead to variable pattern of changes in MMP concentrations, and thus, different dynamics in TBI.

A previous study using western blot methodology was unable to detect CSF levels of MMP-12 in both TBI and iNPH cohorts and CSF levels of MMP-3 and MMP-9 in the iNPH patients^35^. Our method could detect all studied MMPs in CSF, except MMP-13, which showed consistently low levels in all CSF samples. Furthermore, a previous study, investigating MMP levels in multiple sclerosis patients reported that CSF MMP-13 was present only in a small percentage of patients^48^. Moreover, to our knowledge, this is the first report describing human CSF levels of MMP-1, MMP-10 and MMP-12 in relation to TBI.

Lack of correlations between CSF MMP concentrations and trauma severity scoring systems suggest that MMPs might reflect pathologic changes in the brain other than those visualized using standard CT scans. Previously observed lack of association between CSF ECM protein concentrations and injury severity scores in TBI^49^ suggests that ECM-related proteins/enzymes in CSF may act as markers for different pathophysiological processes in TBI than currently used serum protein biomarkers which show good correlations to available CT scoring systems^50^.

Since the MMPs are not CNS specific, extracranial sources could contribute to the total CSF levels through the release of MMPs to CSF following other tissue injuries than brain. However, CSF MMP concentrations in the studied cohort are not influenced by extracranial trauma.

Among the strengths of this study are the well-characterized material based on brain injury biomarker data and clinical examination as well as access to ventricular CSF as EVD is a rare procedure and ventricular CSF probably better reflects brain pathological changes compared with lumbar CSF.

Finally, there are some limitations to the study that should be considered, the most important of which are some missing repeated measures for TBI patients and the lack of ventricular CSF from healthy controls. The study had a mainly exploratory aim, in which it was difficult to address the issue of multiple comparisons. It is also important to point out that MMP CSF levels act as a surrogate for cerebral MMP dynamics and might not express how these proteins are up/down-regulated or expressed throughout the brain. Additionally, the limited sample size prevented us from conducting multivariable analysis, which could confirm if MMPs in CSF are independent outcome predictors in TBI.

In conclusion, this study demonstrates significant changes in the CSF concentrations of several MMPs following TBI. Different MMPs show different patterns in TBI suggesting that individual MMPs have different roles in the pathophysiology following brain injury. CSF MMP-2 and MMP-10 concentrations are able to discriminate clinical outcomes in TBI, similarly as other CSF biomarkers, i.e., S100B and NSE, which makes them promising candidate biomarkers for TBI outcome prognosis.

## Materials and methods

### Ethics

The study was conducted according to the Declaration of Helsinki and ethical approvals were provided by the regional ethical board in Stockholm (#2005/1526/31/2) and in Gothenburg (154-05). Assent or written informed consent was acquired by the patients or next-of-kin.

### Patient characteristics

The study included 33 TBI patients requiring neuro-intensive care prospectively recruited at the Karolinska University Hospital (#2005/1526/31/2) (Table 1). The measurements of consciousness levels, as defined by GCS^6^ assessed at admission to the hospital^25^, indicate that the majority of the patients (73%) suffered from severe TBI (GCS = 3–8) (Table 1), but all were deemed to require neuro-intensive care for their injuries. Additionally, as determined by AIS^8^ assessed using data acquired during the first days of hospital stay^25^, 87% patients were severely or critically injured (AIS = 4–6) (Table 1). GOS was assessed by a questionnaire including key interview questions for GOS at approximately 12 months post injury^25^. In outcome prediction models, GOS scores were commonly dichotomized^15^ into favourable (GOS 4, 5), indicating moderate or full recovery and unfavourable (GOS 1–3) specifying death or severe disability. The cohort did not contain patients with GOS 2, representing persistent vegetative state. Multitrauma was defined according to the Advanced Trauma Life Support (ATLS) guidelines, and indicated significant extracranial trauma as previously described^51^. In the TBI cohort, CSF samples were collected through external ventricular drains (EVD) inserted in either the lateral ventricles or the third ventricle at three time points (TP) after TBI: TP 1 (1–6 days), TP 2 (5–10 days) and TP 3 (7–14 days) (Table 3). Samples were centrifuged for 15 min at 2000*g*, aliquoted and stored at − 80 °C. The samples underwent a total of two freeze–thaw cycles.

**Table 3:** CSF MMP concentrations between TBI (three time points) and iNPH groups.

MMP concentration median (interquartile interval)	TBI			iNPH
	TP 1 (n = 25)	TP 2 (n = 22)	TP 3 (n = 14)	
MMP-1 (pg/mL)	162 (22.9–365)	22.8 (9.16–67.3)	39.0 (11.2–221)	6.31 (4.19–12.2)
MMP-2 (pg/mL)	2820 (617–12,300)	2860 (806–5,720)	2750 (1210–10,600)	7830 (545–10,800)
MMP-3 (pg/mL)	2300 (126–469)	349 (89–640)	297 (110–753)	202 (134–278)
MMP-9 (pg/mL)	246 (127–719)	221 (99.1–463)	179 (95.2–334)	24.8 (19.4–39.7)
MMP-10 (pg/mL)	179 (10.6–268)	35.3 (8.48–64.3)	41.9 (18.6–93.5)	8.17 (6.10–11.9)
MMP-12 (pg/mL)	9.32 (6.10–26.2)	12.3 (6.10–30.9)	17.9 (6.10–33.3)	6.10 (6.10–14.1)

*iNPH* idiopathic normal pressure hydrocephalus, *MMP* matrix metalloproteinase, *TP* time point, *TBI* traumatic brain injury.

For ethical reasons, ventricular CSF is not possible to acquire from healthy individuals; instead 38 patients with iNPH were included in the study as a contrast group. Here, CSF samples were collected through a catheter entered into the right lateral ventricle at the time for shunt surgery.

### Biochemical analyses

MMPs were quantified using two Milliplex MAP Human MMP magnetic bead panels, HMMP1MAG-55K and HMMP2MAG-55K (EMD Millipore Corp., Billerica, MA, USA), following the manufacturer’s instructions. The human MMP Magnetic Bead panel 1 (HMMP1MAG-55K) was used for simultaneous quantification of MMP-3, MMP-12 and MMP-13, while MMP Magnetic Bead panel 2 (HMMP2MAG-55K) measures MMP-1, MMP-2, MMP-7, MMP-9 and MMP-10. In brief, calibrators/quality controls/CSF samples, all diluted 1:2 in assay buffer, were incubated with antibody-magnetic beads mix on the pre-washed 96-well plate for 2 h. After repeated washes of the plate, biotinylated detection antibody was added to the plate and incubated for 1 h, followed by the addition of streptavidin–phycoerythrin concentrate for 30 min. After repeated washes of the plate, the beads were resuspended on a plate shaker using drive fluid (Luminex Corp., Austin, TX, USA) for min 5 min prior to reading. All washing steps were performed with the wash buffer using an automated magnetic 96-well plate washer (Bio-Plex Pro Wash Station, Bio-Rad, Mississauga, ON, Canada). All incubations were performed in the dark at room temperature on a plate shaker (600 rpm). The fluorescence was read in a Luminex Magpix xMAP 200 instrument (Luminex Corp., Austin, TX, USA) and analysed with the Luminex xPONENT Software version 4.2 (Luminex Corp., Austin, TX, USA).

The assays for the detection of NFL, S100B and NSE in CSF have been previously described^25^.

The data was not blinded to the researchers. The repeatedly measured samples, collected at different TPs from the same individual, were analysed adjacent to each other to reduce the within-subject variation of the measurements. The contrast samples were randomly placed across a plate to reduce the impact of spatial systematic errors. All samples were run in duplicates.

Two quality controls (with low and high MMP concentrations) provided in the kits (EMD Millipore Corp., Billerica, MA, USA) were run in duplicates at the beginning and end of each plate. The analytical coefficients of variation (CV) of quality controls were ≤ 12% for repeatability and ≤ 15% for intermediate precision in all measured analytes.

Samples having a low net Median Fluorescent Intensity (MFI), below the limit of quantification, or samples having a high MFI, above the highest calibration point, were replaced with the values of the lowest or highest calibration point, respectively.

### Statistical analyses

Analysis of covariance was used to examine the differences between the two independent groups, such as iNPH and TBI groups as well as favourable and unfavourable outcome groups, accounting for the effect of age. The differences between the longitudinal measurements obtained from TBI patients were analysed using linear mixed effects model. Repeated MMP measures were included in the model as dependent variables, time as fixed factor, individuals as random factors and age as a covariate. The selection of the covariance matrix in the mixed model analysis was made using Akaike Information Criterion (AIC) index. Correlations were assessed using Spearman’s rank correlation. Receiver Operating Characteristic (ROC) analysis was performed to predict unfavourable outcome of TBI patients based on the CSF MMP concentrations at baseline and to evaluate their prognostic ability when compared with other brain injury biomarkers (NFL, S100B and NSE). Areas under the curve (AUC) together with sensitivities and specificities were obtained as measures of performance for the tests.

All statistical analyses were performed using GraphPad Prism, version 7.03 (GraphPad Software, Inc., San Diego, CA, USA) and SPSS software, version 26 (IBM Corp., Armonk, NY, USA). All tests were two-sided and the probability of p ≤ 0.05 was considered statistically significant.

## Supplementary information

Supplementary Information.

## Data Availability

The data supporting the findings in this study are available from the corresponding author, upon reasonable request.

## References

[1] Scholten AC, Haagsma JA, Panneman MJ, van Beeck EF, Polinder S (2014). Traumatic brain injury in the Netherlands: Incidence, costs and disability-adjusted life years. PLoS ONE.

[2] Gubata ME (2014). Trends in the epidemiology of disability related to traumatic brain injury in the US Army and Marine Corps: 2005 to 2010. J. Head Trauma Rehabil..

[3] Dewan MC (2018). Estimating the global incidence of traumatic brain injury. J. Neurosurg..

[4] Blennow K (2016). Traumatic brain injuries. Nat. Rev. Dis. Primers.

[5] Becker DP (1977). The outcome from severe head injury with early diagnosis and intensive management. J. Neurosurg..

[6] Teasdale G, Jennett B (1974). Assessment of coma and impaired consciousness. A practical scale. Lancet.

[7] Starmark JE, Stalhammar D, Holmgren E (1988). The Reaction Level Scale (RLS85). Manual and guidelines. Acta Neurochir. (Wien).

[8] Gennarelli TA, Wodzin E (2006). AIS 2005: A contemporary injury scale. Injury.

[9] Marshall LF (1992). The diagnosis of head injury requires a classification based on computed axial tomography. J. Neurotrauma.

[10] Maas AI, Hukkelhoven CW, Marshall LF, Steyerberg EW (2005). Prediction of outcome in traumatic brain injury with computed tomographic characteristics: A comparison between the computed tomographic classification and combinations of computed tomographic predictors. Neurosurgery.

[11] Raj R, Siironen J, Skrifvars MB, Hernesniemi J, Kivisaari R (2014). Predicting outcome in traumatic brain injury: Development of a novel computerized tomography classification system (Helsinki computerized tomography score). Neurosurgery.

[12] Nelson DW (2010). Extended analysis of early computed tomography scans of traumatic brain injured patients and relations to outcome. J. Neurotrauma.

[13] Firsching R (2001). Classification of severe head injury based on magnetic resonance imaging. Acta Neurochir. (Wien).

[14] Steyerberg EW (2008). Predicting outcome after traumatic brain injury: Development and international validation of prognostic scores based on admission characteristics. PLoS Med.

[15] Jennett B, Bond M (1975). Assessment of outcome after severe brain damage. Lancet.

[16] Sykova E, Nicholson C (2008). Diffusion in brain extracellular space. Physiol. Rev..

[17] Cox TR, Erler JT (2011). Remodeling and homeostasis of the extracellular matrix: Implications for fibrotic diseases and cancer. Dis. Model Mech..

[18] Zetterberg H (2006). Neurochemical aftermath of amateur boxing. Arch. Neurol..

[19] Neselius S (2012). CSF-biomarkers in Olympic boxing: Diagnosis and effects of repetitive head trauma. PLoS ONE.

[20] Franz G (2003). Amyloid beta 1–42 and tau in cerebrospinal fluid after severe traumatic brain injury. Neurology.

[21] Zemlan FP (2002). C-tau biomarker of neuronal damage in severe brain injured patients: Association with elevated intracranial pressure and clinical outcome. Brain Res..

[22] Ost M (2006). Initial CSF total tau correlates with 1-year outcome in patients with traumatic brain injury. Neurology.

[23] Goyal A (2013). S100b as a prognostic biomarker in outcome prediction for patients with severe traumatic brain injury. J. Neurotrauma.

[24] Berger RP (2002). Neuron-specific enolase and S100B in cerebrospinal fluid after severe traumatic brain injury in infants and children. Pediatrics.

[25] Al Nimer F (2015). Comparative assessment of the prognostic value of biomarkers in traumatic brain injury reveals an independent role for serum levels of neurofilament light. PLoS ONE.

[26] Shahim P (2016). Serum neurofilament light protein predicts clinical outcome in traumatic brain injury. Sci. Rep..

[27] Thelin EP (2016). Utility of neuron-specific enolase in traumatic brain injury; relations to S100B levels, outcome, and extracranial injury severity. Crit. Care.

[28] Vos PE (2010). GFAP and S100B are biomarkers of traumatic brain injury: An observational cohort study. Neurology.

[29] Khokha R, Murthy A, Weiss A (2013). Metalloproteinases and their natural inhibitors in inflammation and immunity. Nat. Rev. Immunol..

[30] Zhang H, Adwanikar H, Werb Z, Noble-Haeusslein LJ (2010). Matrix metalloproteinases and neurotrauma: Evolving roles in injury and reparative processes. Neuroscientist.

[31] Gurney KJ, Estrada EY, Rosenberg GA (2006). Blood–brain barrier disruption by stromelysin-1 facilitates neutrophil infiltration in neuroinflammation. Neurobiol. Dis..

[32] Asahi M (2001). Effects of matrix metalloproteinase-9 gene knock-out on the proteolysis of blood–brain barrier and white matter components after cerebral ischemia. J. Neurosci..

[33] Cossins JA (1997). Enhanced expression of MMP-7 and MMP-9 in demyelinating multiple sclerosis lesions. Acta Neuropathol..

[34] Zheng K (2013). Matrix metalloproteinases and their tissue inhibitors in serum and cerebrospinal fluid of patients with moderate and severe traumatic brain injury. Neurol. India.

[35] Grossetete M, Phelps J, Arko L, Yonas H, Rosenberg GA (2009). Elevation of matrix metalloproteinases 3 and 9 in cerebrospinal fluid and blood in patients with severe traumatic brain injury. Neurosurgery.

[36] Abdul-Muneer PM, Pfister BJ, Haorah J, Chandra N (2016). Role of matrix metalloproteinases in the pathogenesis of traumatic brain injury. Mol. Neurobiol..

[37] Fingleton B (1864). Matrix metalloproteinases as regulators of inflammatory processes. Biochim. Biophys. Acta Mol. Cell Res..

[38] Rosenberg GA (2002). Matrix metalloproteinases in neuroinflammation. Glia.

[39] Akai K, Uchigasaki S, Tanaka U, Komatsu A (1987). Normal pressure hydrocephalus. Neuropathological study. Acta Pathol. Jpn..

[40] Tullberg M, Rosengren L, Blomsterwall E, Karlsson JE, Wikkelso C (1998). CSF neurofilament and glial fibrillary acidic protein in normal pressure hydrocephalus. Neurology.

[41] Sternlicht MD, Werb Z (2001). How matrix metalloproteinases regulate cell behavior. Annu. Rev. Cell Dev. Biol..

[42] Ahmed SH (2006). Matrix metalloproteinases/tissue inhibitors of metalloproteinases: Relationship between changes in proteolytic determinants of matrix composition and structural, functional, and clinical manifestations of hypertensive heart disease. Circulation.

[43] Niebroj-Dobosz I, Janik P, Sokolowska B, Kwiecinski H (2010). Matrix metalloproteinases and their tissue inhibitors in serum and cerebrospinal fluid of patients with amyotrophic lateral sclerosis. Eur. J. Neurol..

[44] Brew K, Nagase H (1803). The tissue inhibitors of metalloproteinases (TIMPs): An ancient family with structural and functional diversity. Biochim. Biophys. Acta.

[45] Hamze AB (2007). Constraining specificity in the N-domain of tissue inhibitor of metalloproteinases-1; gelatinase-selective inhibitors. Protein Sci..

[46] Pizzi MA, Crowe MJ (2007). Matrix metalloproteinases and proteoglycans in axonal regeneration. Exp. Neurol..

[47] Nakamura H (2000). Brevican is degraded by matrix metalloproteinases and aggrecanase-1 (ADAMTS4) at different sites. J. Biol. Chem..

[48] Castellazzi M (2018). Multiplex matrix metalloproteinases analysis in the cerebrospinal fluid reveals potential specific patterns in multiple sclerosis patients. Front. Neurol..

[49] Minta K (2019). Dynamics of extracellular matrix proteins in cerebrospinal fluid and serum and their relation to clinical outcome in human traumatic brain injury. Clin. Chem. Lab. Med..

[50] Thelin E (2019). A serum protein biomarker panel improves outcome prediction in human traumatic brain injury. J. Neurotrauma.

[51] Kortbeek JB (2008). Advanced trauma life support, 8th edition, the evidence for change. J. Trauma.

