# Supplementary material for: Dynamics of cerebrospinal fluid levels of matrix metalloproteinases in human traumatic brain injury
Source: Sci Rep. 2020 Oct 22;10:18075. doi: 10.1038/s41598-020-75233-z (PMC7582923; doi:10.1038/s41598-020-75233-z)
Supplement: Supplementary file 1 — Supplementary Information. [file 41598_2020_75233_MOESM1_ESM.pdf]

Dynamics of cerebrospinal fluid levels of matrix metalloproteinases in human traumatic brain injury.

Karolina Minta[1,*], Gunnar Brinkmalm[1,2], Faiez Al Nimer[3], Eric P. Thelin[3,4], Fredrik Piehl[3], Mats Tullberg[5], Anna Jeppsson[5], Erik Portelius[1,2], Henrik Zetterberg[1,2,6,7], Kaj Blennow[1,2], Ulf Andreasson[1,2]

[1]Department of Psychiatry and Neurochemistry, Institute of Neuroscience and Physiology, the Sahlgrenska Academy at the University of Gothenburg, Sweden [2]Clinical Neurochemistry Laboratory, Sahlgrenska University Hospital, Mölndal, Sweden [3]Department of Clinical Neuroscience, Karolinska Institutet, Stockholm, Sweden [4]Department of Neurology, Karolinska University Hospital, Stockholm, Sweden [5]Department of Clinical Neuroscience, Institute of Neuroscience and Physiology, the Sahlgrenska Academy at the University of Gothenburg, Sweden [6]Department of Neurodegenerative Disease, UCL Institute of Neurology, London, UK [7]UK Dementia Research Institute at UCL, London, UK

*Corresponding author:

Karolina Minta

Department of Psychiatry and Neurochemistry

Sahlgrenska University Hospital/Mölndal,

S-431 80 Mölndal, Sweden

e-mail: karolina.minta@neuro.gu.se

tel: +46735660741

Supplementary figure 1. Association between MMPs in CSF and multitrauma in TBI patients (at time point 1).

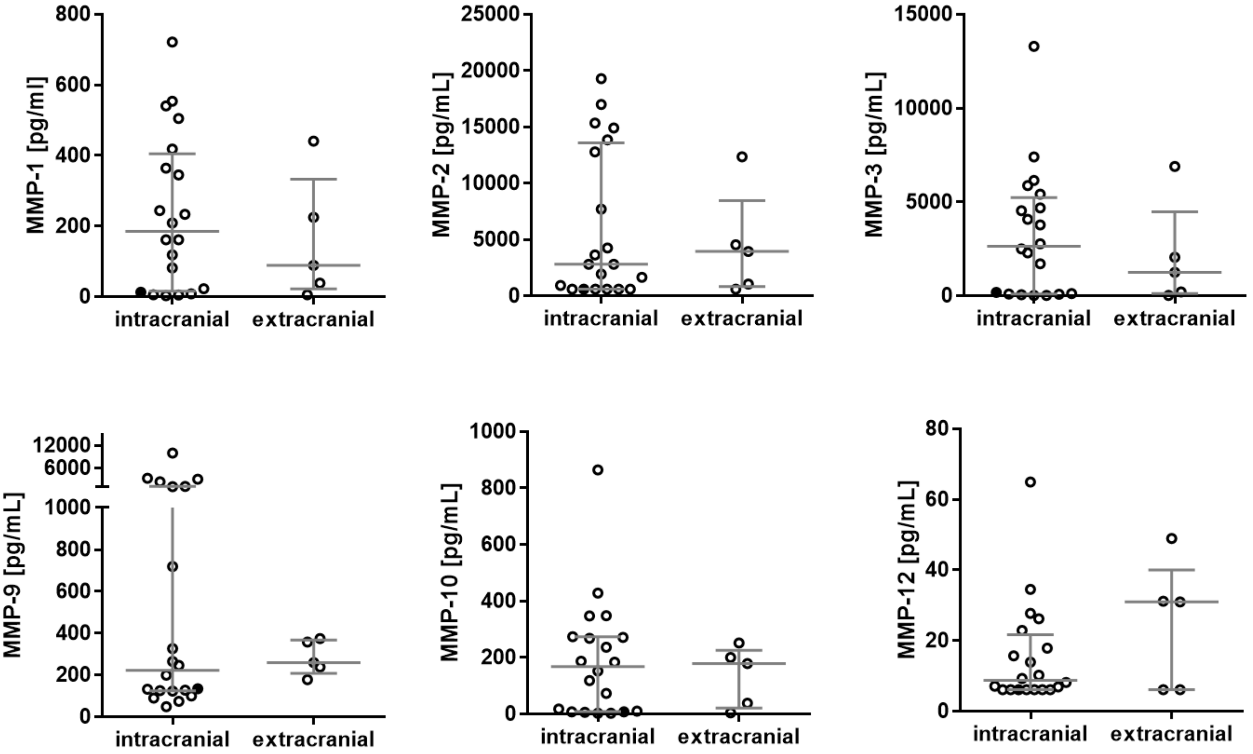

The horizontal lines represent the median and interquartile ranges.

N (number of patients): n=20 for intracranial injuries, n=5 for extracranial injuries
